# Chromoblastomycosis Caused by Phialophora verrucosa in a Costa Rican Child with Skin Sequelae due to Snake Bite

**DOI:** 10.7759/cureus.3574

**Published:** 2018-11-12

**Authors:** Helena Brenes, Marco L Herrera, María L Ávila-Aguero

**Affiliations:** 1 Pediatrics, Hospital Nacional De Niños "dr. Carlos Sáenz Herrera" Caja Costarricense Del Seguro Social, San Jose, CRI; 2 Bacteriology, Hospital Nacional De Niños "dr. Carlos Sáenz Herrera" Caja Costarricense Del Seguro Social, San jose, CRI

**Keywords:** chromoblastomycosis, snake bite sequelae, phialophora verrucosa

## Abstract

Chromoblastomycosis is an implantation mycosis occurring among adults working in farms or with soil in tropical and subtropical areas worldwide. *Fonsecaea pedrosoi* is the most important agent in the tropical areas, while *Phialophora verrucosa*, although not a predominant agent, is found in the lowlands under the same conditions as the *Fonsecae* species.

We present the case of a 10-year-old aboriginal boy, belonging to a soil worker family, with a history of extensive leg lesions and lymphedema secondary to a snake bite five years earlier. He was admitted to the National Children's Hospital (part of the Caja Costarricense del Seguro Social: the social security system in Costa Rica) with multiple verrucous confluent lesions on the ankle, some with dark coloration, and no other symptoms. Clinical suspicion of chromoblastomycosis was made and later confirmed by culture. Itraconazole was started showing clinical improvement.

Chromomycosis, especially in the population with skin lesions or chronic tissue compromise, associated with the location and macroscopic findings, must be a part of our differential diagnosis. The story of an exposed pediatric patient to soil work and the history of an important leg swelling and skin disruption as sequelae of snake bite envenomation made this case unique. To our knowledge, there are no pediatric reports of Chromoblastomycosis in Latin America.

## Introduction

Chromoblastomycosis, first described in Brazil in 1911 by Moraes Pedroso [1], is an implantation mycosis occurring among adults working in farms or with soil in tropical and subtropical areas worldwide, particularly in the Central American countries. It is a chronic human melanized fungal infection of the subcutaneous tissues caused by the traumatic inoculation of a specific group of dematiaceous fungi through the skin (usually *Fonsecaea pedrosoi*, *Phialophora* or *Cladophialophora carrionii*) [2]. These agents are slow-growing fungi with low virulence and high tolerance to heat [1]. The agents causing chromoblastomycosis are common soil saprophytes. *F*.* pedrosoi *is the most important agent in the tropical areas, while *P. verrucosa*, although not a predominant agent, is found in the lowlands under the same conditions as the Fonsecaea species [1].

Differential diagnoses may include infectious diseases, such as paracoccidioidomycosis, leishmaniasis and verrucous tuberculosis, and non-infectious disorders, including sarcoidosis and psoriasis [3].

The first case of chromomycosis in Costa Rica was diagnosed in 1928, and by the 1950s, this became an endemic country [4]. However, due to the improvements in the living conditions and social development, these cases are currently extremely rare. Nevertheless, Costa Rica is a tropical country and has optimal weather and soil conditions favorable for fungi; hence, clinical suspicion should be maintained.

## Case presentation

A 10-year-old aboriginal boy, belonging to a soil-working family, came from Panama with his relatives to collect the coffee harvest, as done every year. In 2010, he was admitted in a Panamanian hospital after a snake bite, with tissue compromise that required a prolonged hospitalization of approximately six months besides multiple skin grafts.

After this hospitalization, he developed a chronic lymphedema in the right leg, with partial function limitation, but did not seek medical attention at any center. In December 2013, they traveled to Costa Rica where he was seen and referred for clinical evaluation to our center, the only pediatric tertiary referral center in the country (part of the Caja Costarricense del Seguro Social: the social security system in Costa Rica). Because of his extensive lesion and lymphedema, he was hospitalized for diagnosis and treatment. 

**Figure 1:**
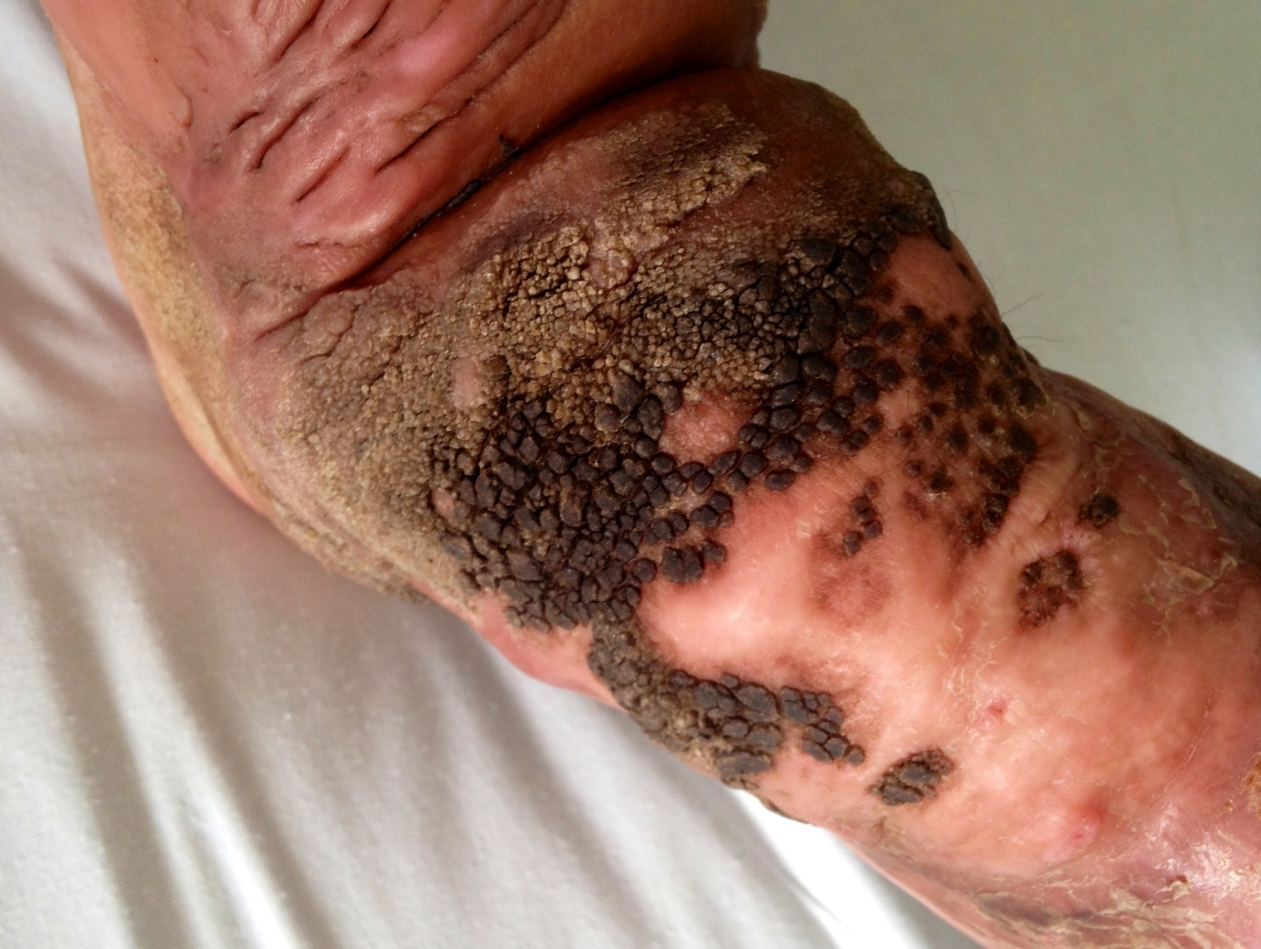
Clinical presentation at admission It shows leg changes secondary to infection at admission. The associated verrucous lesions and pigment changes are evident.

On admission, we document a eutrophic child, without a history of fever, some skin lesions suggestive of scabies, no cardiopulmonary compromise, and no pathological abdomen findings. The patient presented with lymphedema in the right leg and foot, with hypertrophy of the skin, edema and inflammatory changes, without compromising the pulse rate. There was evidence of multiple verrucous confluent lesions, a few with dark coloration, on the ankle (Figure 1).

On admission, his early laboratory report showed no anemia (hemoglobin 14.2 g/dl, 39% hematocrit), white blood cell and differential count of 8360 cells/mm, with normal leucocytes, eosinophilia (2508 cells/mm) and normal platelets (332,000 cells/mm). Urinalysis, blood urea nitrogen (BUN) test, tests measuring the levels of liver enzymes, including aspartate aminotransferase (AST) and alanine aminotransferase (ALT), and albumin test yielded normal results (BUN 11 mg/dl, creatinine 0.4 mg/dl, AST 33 U/l, ALT 21 U/l, albumin 3.8 g/dl). Radiographs of the affected limb showed no bone involvement, with soft tissue inflammation only.

An infectious disease specialist was consulted, and the clinical suspicion of chromoblastomycosis was made. Skin lesions were examined using smears and cultures of dermal scrapings, and sections obtained from skin biopsy were also studied. Oral itraconazole was initiated. and because of clinical suspicion of secondary cutaneous bacterial superinfection, intravenous clindamycin and cefotaxime were administered for seven days, with negative bacterial cultures.

**Figure 2:**
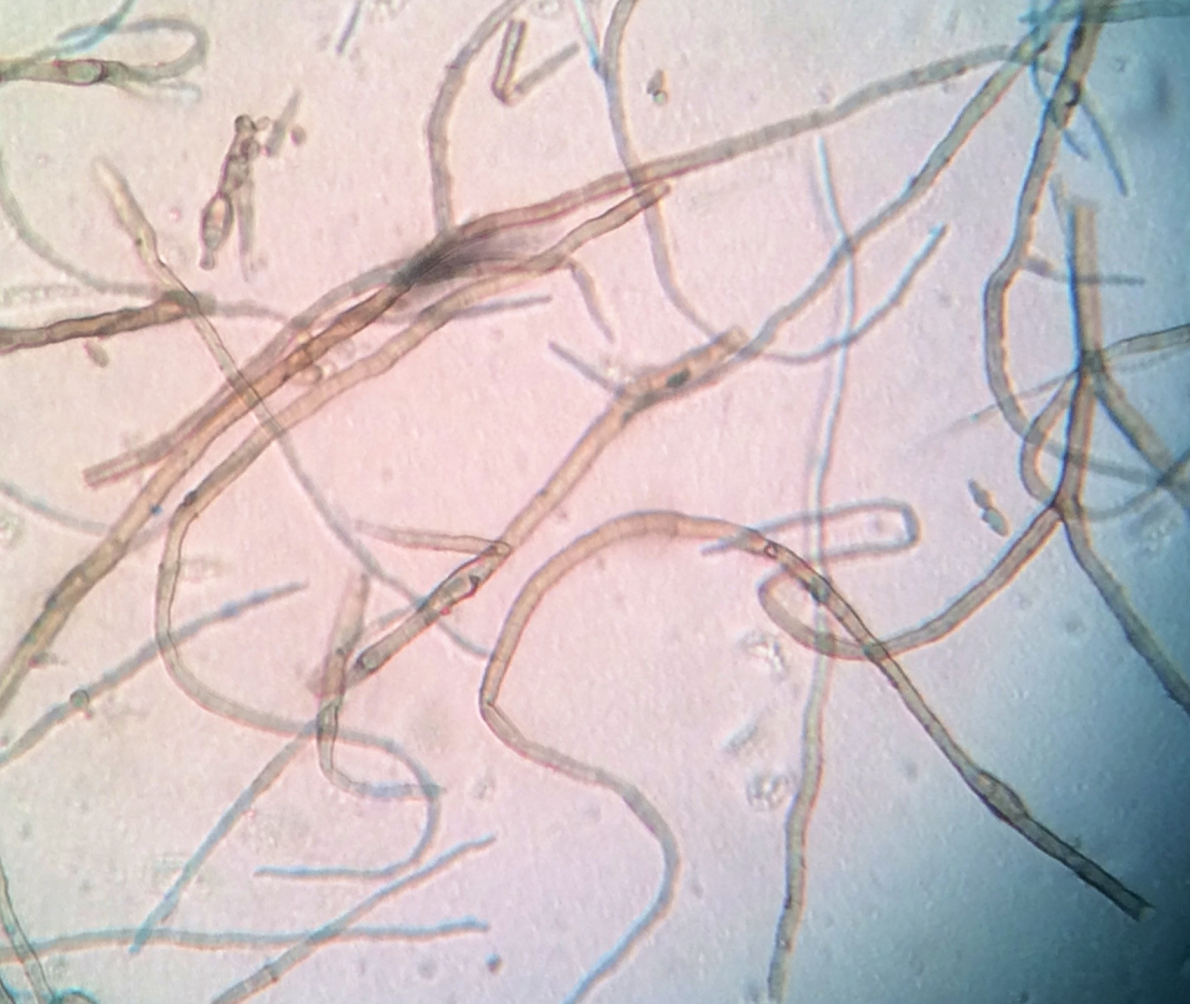
Microscopic findings of biopsy samples Presence of hypha forms, suggesting diagnosis

The clinical diagnosis of chromomycosis in this patient was confirmed with direct examination with potassium hydroxide (KOH), documenting the presence of hypha-forms, with fungal culture in Sabouraud's agar positive for *P. verrucosa* (Figure 2).

**Figure 3:**
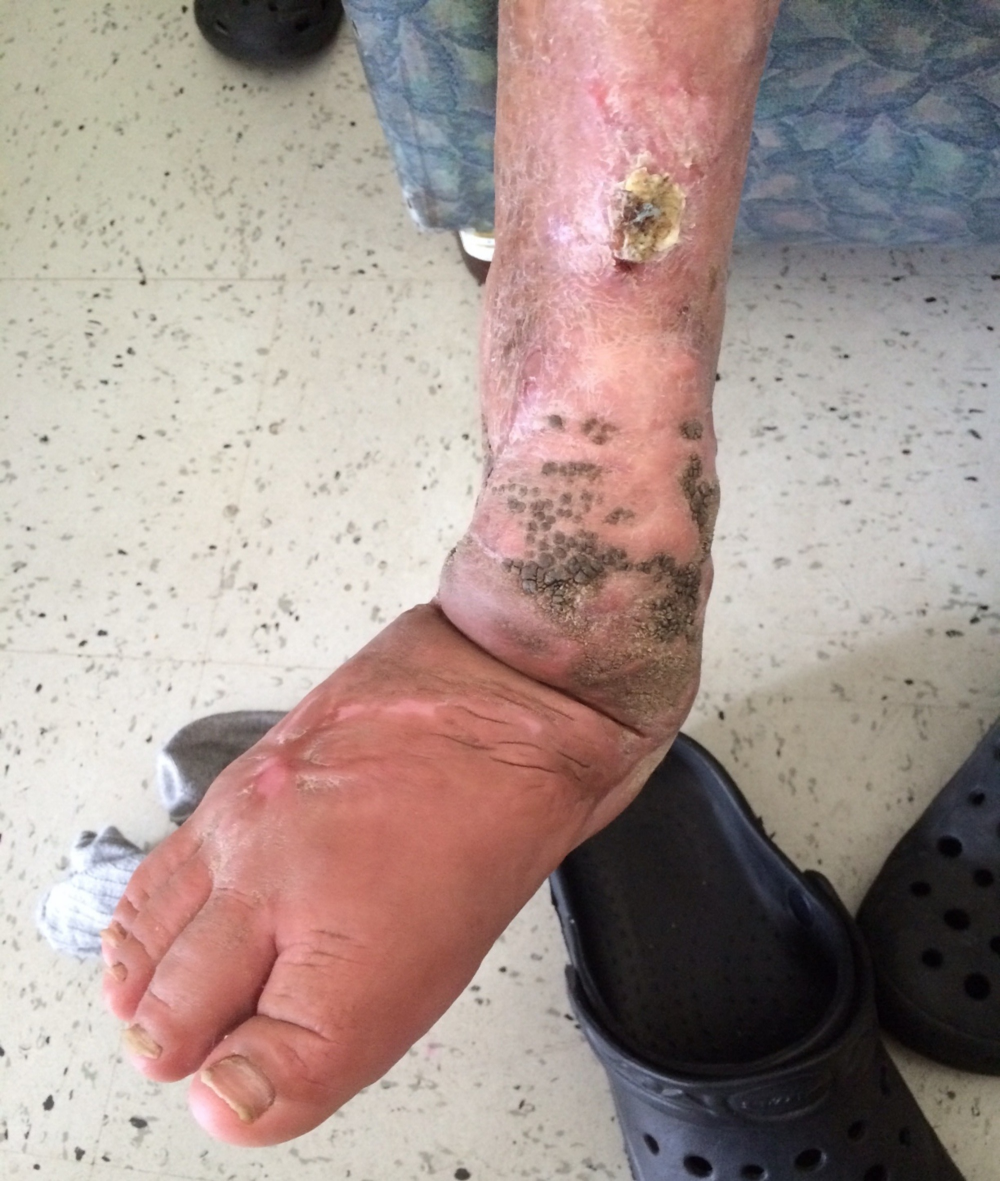
Clinical evolution before discharge

After a 22-day oral treatment with itraconazole, there was a clinical improvement without functional compromise, and *P. verrucosa* lesions were less evident (Figure 3).

## Discussion

Lymphedema is a chronic progressive swelling of tissues caused by lymphatic vessel dysfunction [5]. In this patient, lymphedema was evident, and chronic changes were apparent on the skin. Lymphedema of extremities, especially in the pediatric population, has different possible diagnoses, including lymphatic malformations [5], rheumatologic disease, vascular compromise such as hemangioma or vasculitis [5].

One of the most important aspects of this patient is the history of a traumatic event (snake bite), with important sequelae that required skin grafts and prolonged medical care. When there is extensive tissue compromise in snake bite, it is likely to associate lymphatic compromise [5] and, in our patient, there is also history that this swelling process occurred secondary to the event. However, it was evident that not only lymphedema but also the presence of verrucous skin lesions could not be explained by the patient's previous medical history. When this patient was taken to the operation room, the skin samples were taken for biopsy and microscopic analysis, documenting papillomatosis and hyperkeratosis, with lymphocyte infiltration and fibrosis. The hypha-forming activity suggested the presence of a black yeast fungal infection.

Chromoblastomycosis is difficult to treat and is often refractory to different therapeutic approaches. The use of itraconazole has shown improvement after a prolonged treatment of more than six months. Sometimes, a long-term continuous treatment or even monthly pulses are necessary to prevent relapses of the disease [1,6]. As shown in the images, our patient had good outcomes after the first three weeks of treatment, probably associated to the close follow-up and clean up made in the operating room, but a total healing was not expected after a long-term antifungal course.

When someone suspects implantation mycosis, we normally consider farmers or soil workers [7-10], with many publications of fungal infections in adults. But the story of a pediatric patient exposed to soil work with a history of a significant leg swelling and skin disruption due to a previous event makes this case unique. In fact, to our knowledge, this is the first pediatric report of Chromoblastomycosis in Latin America.

Chromomycosis, especially in the population with skin lesions or chronic tissue compromise, associated with the location and macroscopic findings, must be part of our differential diagnosis [11].

## Conclusions

Implantation mycosis is normally found in specific populations working on farms or soil. Several such studies are available in adults but are uncommon in the pediatric patients. Although there was a history of a traumatic event that could explain part of the tissue compromise in our patient, after a multidisciplinary and complete investigation on the patient, we were able to document a secondary mycotic infection with a favorable outcome once treatment was administered.
